# “Those fires I had to work through was a learning”: An evaluation of the Eeyou Istchee Daily Smoke Monitoring Report

**DOI:** 10.14745/ccdr.v52i78a08

**Published:** 2026-07-30

**Authors:** Anna JW Manore, Jason Coonishish, Isabelle Duguay, Colleen Fuller, Juliette Ottereyes, Environmental Health Team

**Affiliations:** 1 Public Health Department, Cree Board of Health and Social Services of James Bay, Mistissini, QC; 2 Canadian Field Epidemiology Program, Centre for Emergency Preparedness, Public Health Agency of Canada, Montréal, QC; 3 Emergency Measures and Disaster Planning, Cree Board of Health and Social Service of James Bay, Chisasibi, QC; 4 Office of Fire Protection, Capital Works and Services, Cree Nation Government, Montréal, QC

**Keywords:** environmental health, forest fire, Indigenous health, Cree health, Eeyou Istchee, surveillance evaluation, thematic analysis

## Abstract

**Background:**

In 2023, Eeyou Istchee experienced a record-breaking forest fire season, causing unprecedented damage to interconnected land, ecosystems, and cultural infrastructure. Acute health impacts of forest fires can be severe, including respiratory and cardiovascular problems, and heat-related illness. From May 2023 to September 2023, the Cree Board of Health and Social Services of James Bay (CBHSSJB) shared verbal updates on smoke, fire, and heat at daily meetings with public health and broader emergency response partners. In 2024, CBHSSJB formalized these verbal updates into a daily written report, distributing publicly available information via email to inform a timely public health response.

**Objective:**

To evaluate the usefulness of the *Eeyou Istchee Daily Smoke Monitoring Report* (shortened to the “Report”) following the first season of implementation and identify potential improvements.

**Methods:**

The evaluation was co-developed with key partners using participatory approaches, with evaluation objectives focusing on the Report’s usefulness for decision-making with respect to smoke, fire, and heat. Key informant interviews (n=6) were carried out with Report recipients, and results were summarized using thematic analysis.

**Results:**

Recipients described the Report as useful and a source of reassurance about current conditions in Eeyou Istchee. Suggested improvements included the addition of more visuals, plainer language, and supporting recipients seeking additional information to complement the Report (i.e., more specific than daily updates at a regional level).

**Conclusion:**

Opportunities for improvement identified through this evaluation informed updates to the Report for 2025 and beyond, and may also be applied by other Indigenous organizations developing or enhancing their own environmental monitoring and reporting processes.

## Introduction

“*Since time immemorial, forest fires have always been present in Eeyou Eenou Istchee and across Canada, shaping forests and ecosystems. Our ancestors understood fire as a natural force, one that could both renew and destroy, and they learned how to live with it.*”—Reggie Tomatuk, Planning Programming and Research Officer, Cree Board of Health and Social Services of James Bay Regional Public Health

Severe forest fires have occurred in recent years in Canada and globally (1–5). Despite longstanding Indigenous knowledge, understanding and practices for how to live with fire, recent wildfires have had disproportionate impacts on Indigenous populations for reasons including remote geography and limited firefighting resources (6–9). These impacts are expected to intensify as climate change increases the frequency and severity of wildfires (10–14).

Unprecedented forest fires in 2023 burned 11% of Eeyou Istchee (5,11,15), which is an area of over 400,000 km^2^ located along the east coast of James Bay and inland and home to approximately 20,000 Eeyouch/Eenouch (Cree). Eight of the nine Cree First Nation communities served by the Cree Board of Health and Social Services of James Bay (CBHSSJB) were evacuated due to fire between June 2023 and August 2023, resulting in negative physical, emotional, mental, and spiritual impacts on Eeyouch/Eenouch. Fires and smoke led to intermittent road closures, impacting the movement of people, supplies, and food in and out of communities. Changes to land had both immediate and long-term impacts on territory, wildlife, and plants crucial for hunting, fishing, and other ways of connecting with Cree culture (9,11).

In Canada, wildfire smoke reporting and risk communication are primarily implemented through centralized tools, such as the Air Quality Health Index (AQHI), which translate ambient PM2.5 and co-pollutant measurements into standardized public health guidance. The AQHI may have limited sensitivity to wildfire smoke—specific exposure patterns and local variability during extreme fire events (16,17). This approach is less accessible and contextually appropriate in Indigenous communities, where structural inequities, language differences, and limited integration of Indigenous governance and knowledge systems may reduce the effectiveness of risk communication and protective actions (18). Cree emergency response teams need to make quick, informed decisions about use of remote transportation routes to ensure continuity of community food supplies, use of traplines, and preparation for evacuations of medically vulnerable individuals. Public health response includes liaison roles, coordination of clean air spaces and sheltering in place resources, distribution of N95 masks outside of clinic settings, public health messaging, and surge capacity. During the 2023 fires, the CBHSSJB regional Environmental Health Team was requested to provide daily regional information on smoke, fire, and heat to partners to inform such actions. With no pre-existing surveillance product, team experts reviewed the fragmented data available and used this to provide verbal updates of relevant information.

In 2024, a structured daily report was developed to monitor fire activity, forecast smoke movement across the territory, track air quality measures, and predict maximum heat (*Eeyou Istchee Daily Smoke Monitoring Report*, shortened to the “Report”) (**Appendix**, **Supplemental material**, Figure S1–S2 and Table S1) to improve the delivery and use of this surveillance service. The Report was evaluated for its usefulness in supporting public health action following the first season of implementation. This evaluation directly responds to an expressed need for enhanced environmental monitoring, coordination, and communication during emergencies in Eeyou Istchee (12,19).

With a significant proportion of the population in Eeyou Istchee being particularly vulnerable to the health impacts of smoke, fire, and heat, monitoring with timely communication and coordination between adaptive response partners is crucial for managing a rapid response to the complex needs arising from these increasingly severe events.

## Methods

### Evaluation planning

In keeping with the Miyupimaatisiiun Research Principles of collaborative planning, execution and interpretation, as well as previous work on evaluation in Indigenous contexts (20–22), this evaluation was co-developed with 2024 Report producers and recipients (**Table 1**).

**Table 1:** Producers and recipients of the 2024 and 2025 *Eeyou Istchee Daily Smoke Monitoring Report*

Report producers	2024 report recipients	2025 report recipients^a^
CBHSSJB Environmental Health	CBHSSJB Environmental Health CBHSSJB Emergency Measures CBHSSJB Communications Community Miyupimaatisiiun Centres (CMCs) and Hospitals, as needed Cree Nation Government (CNG) Office of Fire Protection	CBHSSJB Environmental Health CBHSSJB Emergency Measures CBHSSJB Communications Community Miyupimaatisiiun Centres (CMCs) and Hospitals Cree Nation Government (CNG) Office of Fire Protection CNG Regional and Community-based Public Safety Officers CBHSSJB Occupational Health

Abbreviations: CBHSSJB, Cree Board of Health and Social Services of James Bay; CNG, Cree Nation Government

^a^ Recipients were added to the distribution list at varying dates throughout the 2025 forest fire season

Discussion groups identified key questions that were used to develop evaluation objectives. Questions and objectives were mapped to United States Centers for Disease Control and Prevention *Updated Guidelines for Evaluating Public Health Surveillance Systems* attributes reflecting usefulness and data quality (23) (**Table 2**). Usefulness is the degree to which the Report *“contributes to the prevention and control of adverse health-related events”* related to smoke and wildfires, *“including an improved understanding of the public health implications of such events.”* Data quality *“reflects the completeness and validity”* of the information contained in the report. While a broader internal evaluation of the Report also employed a survey, a review of social media messaging, and a comparison of reported and recorded temperatures, this article focuses solely on the assessment of usefulness through qualitative methods.

**Table 2:** Evaluation objectives and corresponding key questions identified by 2024 *Eeyou Istchee Daily Smoke Monitoring Report* producers and recipients

Evaluation objectives	Key question(s)
Contextualize the resources required to produce and distribute the Report	How far did the Report go (i.e., reach)? Would different “levels” of reporting (i.e., shorter reports on quiet or calm days) meet folks’ needs?
Describe actions taken as a result of the Report	How far did the Report go (i.e., reach)?
Describe how the Report was used by those who received it, and how it supported decision-makers in Eeyou Istchee in their response(s) to smoke, fire, and heat	How far did the Report go (i.e., reach)? Does the format serve the intended audience, or could it be improved? Is the frequency of the Report acceptable? Would different “levels” of reporting (i.e., shorter reports on quiet or calm days) meet folks’ needs?
Identify which sections or aspects of the Report are more or less useful to those who received it	Does the Report include the information necessary for decision-makers? Which information in the Report is most useful for decision-makers? Is there any information that’s not needed?

Abbreviation: Report, *Eeyou Istchee Daily Smoke Monitoring Report*

### Key informant interviews

In the summer of 2025, six semi-structured interviews (45–60 minutes each) were conducted with 2024 Report recipients. Invitations for key informant interviews were extended via email, focusing on organizations and communities that responded to heavy smoke or fire in 2024 (the first season of Report implementation). A convenience sample was made of all willing participants. A conversation guide was co-developed with producers and recipients based on the key questions (Appendix, Supplemental material, List S1) and walked through example reports from both 2024 and 2025 (Appendix, Supplemental material, Figure S1–S2). Interviews were conducted via Microsoft Teams, including transcription and recording with prior consent. Transcripts were reviewed by a co-author and shared with key informants for validation.

### Thematic analysis

Thematic analysis was conducted in Microsoft Excel using *a priori* and emergent themes (**Table 3**). These were structured into content themes and action themes. The analysis followed a structured, iterative process involving initial familiarization with the data, systematic coding of themes, identification of emergent themes, and refinement to enhance transparency and analytic rigour. A member of the CBHSSJB Public Health Department served as a second reviewer, coding two anonymized interview transcripts and discussing findings with a co-author to ensure coherence.

**Table 3:** *A priori* and emergent themes employed in thematic analysis of key informant interviews

Evaluation objectives	Origin	Themes
1,2,3,4	Overall aspects of the Report (*a priori*)	Actions informed by the Report Recipients’ priorities within the Report
		Report format and frequency
2,3,4	Sections of the Report (*a priori*)	Air quality
		Temperature
		Wind
		Visibility
		Report recommendations
3,4	Emergent themes	Reporting as reassurance
		Learning and preparedness
		Jurisdictions and coordination

Abbreviation: Report, *Eeyou Istchee Daily Smoke Monitoring Report*

### Ethical considerations

Public health surveillance evaluation falls outside the CBHSSJB definition of research (21). However, in keeping with Miyupimaatisiiun Research Principles (20) and First Nations Principles of ownership, control, access, and possession (OCAP®) (24), the evaluation protocol was discussed with the CBHSSJB Research Office during planning, and the manuscript was reviewed by the Research Advisory Committee (RAC) of the CBHSSJB. Key informants were assured of confidentiality, informed about the evaluation objectives and methods advised how their information would be used, and provided verbal or written consent before participation.

## Results

### Key informant interviews

Interviewees were employees of CBHSSJB (Miyupimâtisîun [n=2], Emergency Measures [n=1], Public Health [n=2]) and the Cree Nation Government Office of Fire Protection (n=1). This represented four of five groups who received the Report in 2024 (i.e., before the distribution list was expanded in 2025).

### Theme: Actions informed by the report

The Report objectives are: 1) to monitor fire, smoke, air quality, and temperature conditions in Eeyou Istchee to support a rapid public health response, and 2) to determine when criteria are met for activation and de-activation of an Activated Public Health Incident Management System (IMS) structure.

Actions potentially informed by the Report varied according to the roles of recipients and the severity of the situation (**Table 4**).

**Table 4:** Actions potentially informed by the *Eeyou Istchee Daily Smoke Monitoring Report*

Evaluation objectives	Theme	Observations
1,2,3,4	Actions potentially informed by the Report	According to key informants, the Report could have been used to inform the following: · Increasing staffing or approving overtime · Increasing environmental monitoring · Conducting public communications · Providing information, reassurance, and support to colleagues and community partners · Rescheduling community activities or services (i.e., garbage collection) · Communicating with outdoor workers to wear masks if air quality is poor · Deploying clean air spaces (i.e., coordinating with Public Safety Officers to block building ventilation systems) · Conducting health risk assessments to support prioritization for potential evacuations · Coordinating the evacuation of vulnerable populations or entire communities (i.e., logistics, identifying vulnerable populations, being in contact with local emergency coordination teams)

Abbreviation: Report, *Eeyou Istchee Daily Smoke Monitoring Report*

Additional results of the thematic analysis are summarized according to key questions in **Table 5**, and by theme in **Table 6**.

**Table 5:** Mapping of objectives and key questions to thematic analysis results

Evaluation objective(s)	Key question	Summary of results
1,2,3	How far did the Report go?	Recipients reported sharing information in the Report with Provincial Civil Security organisations, Community Fire Chiefs, CMC Staff and Clients, Community Emergency Management Committees, and concerned community members.
1,3	Would different “levels” of reporting meet recipients’ needs?	Multiple recipients cited the daily Report as reassuring and indicated a “scaled back” Report on days with no concerns would also meet their needs. One recipient suggested an abbreviated report similar to the 2025 first page on such days.
3	Did the format serve the intended audience, or could it be improved?	Recipients praised the more visual elements of the Report, while opinions on more text-heavy components were mixed. Some recipients expressed a desire for a more summarized version that could be shared more broadly. Receiving the Report as an email generally works. Interviews appreciated that information was delivered directly to them in the morning without having to seek it out, and that it was searchable in their inboxes later in the day.
3	Is the frequency of the Report acceptable?	Generally, yes. Report recipients appreciated the daily update, and twice-daily in case of heightened concerns and/or quickly changing circumstances. However, recipients also described situations where decision-making required information very specific to a given time or location (i.e., beyond the scope of a daily report at the regional level).
4	Does the Report include information necessary for decision-makers in the response to smoke, fire, and heat?	Generally, yes. Suggestions for additional information to add included air quality at locations outside of communities.
4	Which information in the Report is most useful for decision-makers?	Most recipients mentioned smoke and air quality as a priority when reading the report. Other sections identified as priorities were temperature, and recommendations for public health action.
4	Is there any information that’s not needed?	The general sentiment was captured by one interviewee who said, “it’s all interesting and it’s all useful.” However, information on wind and visibility were described as potentially less useful if there are no active fires or heavy smoke in the territory.

Abbreviations: CMC, community Miyupimaatisiiun centre; Report, *Eeyou Istchee Daily Smoke Monitoring Report*

**Table 6:** Summary of thematic analysis results and corresponding opportunities for improvement

Theme	Observation(s)	Opportunities for improvement
Recipients’ priorities within the Report	Air quality was mentioned most often as a priority among recipients, followed by temperature and recommendations for public health action	Continue reporting on air quality and temperature, and providing written recommendations
Format and frequency	Visual elements of 2025 Report were appreciated, while the paragraphs of written text were described as “very lengthy”	Additional visual elements (i.e., maps or infographics); highlighting key words or locations
Format and frequency	Recipients appreciated being able to quickly scan the Report for the name of their own and neighbouring communities; however, Eeyouch/Eenouch often live and travel outside of communities	Include locations beyond communities; prioritise a more regional summary
Format and frequency	The morning timing of the Report was useful for planning the workday; important to have information on smoke, fire, and heat on hand for daily coordination meetings	Continue daily (morning) reporting, and twice-daily reporting as appropriate
Format and frequency	Recipients appreciate daily reporting, and there were no objections to a scaled-back report on days with no concerns	Continue daily reporting; consider sending out only the 2025 first page on days where there are no concerns
Air quality	Current and future smoke and air quality were critical for contextualising health risks and informing public health interventions (e.g., determining sites to receive evacuees)	Continue reporting on both current and future smoke and air quality
Temperature	Information on temperature informed the public health response to heat, and to compound hazards of heat and smoke	Continue reporting on temperature; adapt recommendations for heat when both smoke and heat are present
Wind	Wind information was useful due to its influence on smoke and fire behaviour, but is potentially less relevant if there are no fires or smoke to be impacted	Report on wind only if there are major threats to a community from fire or smoke; support recipients in finding trusted information on wind specific to their situation
Visibility	Visibility can be critical information in determining if road or air travel between communities is possible (i.e., for evacuations or resupply), but is potentially less relevant when air quality is good	Report on visibility only if there are major threats to a community from fire or smoke; support recipients in finding trusted information on visibility specific to their situation
Report recommendations	Recommendations in the Report guided recipients’ conversations with community partners and participation in emergency response meetings	Continue providing written recommendations in the Report
Report recommendations	Recipients appreciated recommendations tailored to specific audiences (e.g., daycares, workplaces, event planners), communities, or buildings, and incorporating Cree traditional knowledge	Continue tailoring recommendations to reflect context-specific audiences and realities
Reporting as reassurance	Information on smoke and forest fires was a source of reassurance for multiple recipients, about their own communities and neighboring communities where they, their colleagues, or patients may have loved ones	Continue daily reporting including a regional overview to all recipients
Jurisdictions and coordination	Interviews underscored the importance of clear roles and responsibilities among partners in emergencies related to smoke, fire, and heat	Collaborate with partners to clarify roles and responsibilities via discussions, tabletop exercises, or other means as appropriate in advance of future forest fire seasons; where possible, indicate a responsible party or parties when providing written recommendations
Learning and preparedness	Previous experiences with forest fires were a source of learning that could be applied to future forest fire seasons	Reflect on lessons learned and apply them to future forest fire preparedness activities; host pre-season discussions or training sessions

Abbreviation: Report, *Eeyou Istchee Daily Smoke Monitoring Report*

### Report priorities identified by recipients

Air quality was mentioned as a priority in five interviews, followed by temperature and recommendations for public health action (three each).

### Theme: Format and frequency

All key informants praised the visual elements of the Report (i.e., maps) for being quick to read and easy to share. The Report often refers to specific communities, which recipients appreciated being able to quickly scan for their own community, or communities where they or their colleagues have loved ones. However, this format creates an information gap for health risks to Eeyouch/Eenouch on the land or highways (i.e., outside of communities). Sending the Report by email in the morning made it easy to check at the beginning of the workday, easy to search for later, and saved time by eliminating the need to seek information elsewhere.

While recipients appreciated daily reporting, a scaled-back Report on days when air quality and temperature are below thresholds of concern would also fulfill this need. One recipient suggested sending out only the first page of the 2025 Report on such days (i.e., a map of current air quality and a brief summary of anticipated air quality and temperature).

Twice-daily reporting was appreciated when recommendations were particularly time-sensitive (e.g., closing windows during the day or overnight) or anticipated to change throughout the day. Recipients noted the importance of having information readily available for emergency response meetings.

### Theme: Air quality and temperature content

Both air quality and temperature were noted as priority topics within the Report as this information was used to respond to health risks from smoke and heat. When communities are under threat of evacuation, the smoke forecast was described as “critical” in determining appropriate sites to receive evacuees. One interviewee described an experience from 2023 (prior to the written Report):

“For [COMMUNITY A], there was a second evacuation. And we evacuated to the sister community next door, which is [COMMUNITY B], and that really helped making those decisions. I was kind of worried that the community we had chosen at first, but then once we had this report, [COMMUNITY B] only had that smoke for maybe an hour, and then the wind shifted and they were OK for about two or three days.”

Temperature information informed decision-making around potential health hazards and fire behavior:

“If we had an emergency and if the temperature is kind of humid and cold, the fire slows down. So, it gives you those hours to work on something.”

### Theme: Wind and visibility content

Wind and visibility information were useful when they impacted existing smoke and fire(s) and informed evacuation and travel decisions during emergencies:

“If there's no visibility, you can't get people out.”

“We had a window of maybe three or four hours to the wind was going to shift so the highway would be accessible to go home. And we had to plan a convoy to go up with the 18-wheelers so they can bring in food, gas and also bring in the people that went to a, to a hockey tournament. There was […] people stuck in [COMMUNITY] that wanted to go home.”

### Theme: Report recommendations content

Report recommendations informed conversations with colleagues and community partners, and guided recipients’ participation in emergency response meetings:

“People always want a written recommendation.”

“[At] local emergency team meetings, that's the first thing they ask: What are the recommendations from Cree Health Board?”

Recommendations tailored to specific audiences (e.g., daycares, work places, event planners) were also appreciated, with daycares in particular as a key audience since closures can have widespread impacts in a community. Recipients also suggested tailoring recommendation to reflect the realities of buildings or infrastructure in Eeyou Istchee (e.g., preventing smoke from entering gaps in windows or doors), and incorporating or acknowledging Cree traditional knowledge:

“Traditionally they would just use a cloth, and they would wet it with water, and it would be the filter for smoke. Again, I guess it comes back to traditional ways of doing things that were also comforting and reassuring."

### Emergent theme: Reporting as reassurance

Information on smoke and forest fires from the Report was a source of reassurance for recipients regarding their own community and neighboring communities where they, their colleagues, or patients may have loved ones:

“When there's fires within our region, whether they be in [COMMUNITY] or…. We have a lot of the community, there's family there, you know, so they do, they do worry. And so, to ease their worry, I call a meeting… So, I just update them where so they can concentrate on work where they don't have to think about, OK, my family over there, I wonder how they're doing.”

### Emergent theme: Jurisdictions and coordination

Recipients emphasized the importance of clear roles and responsibilities in emergency situations, and a desire for clarity around who would be responsible for carrying out the recommendations provided in the Report (e.g., communicating with partners and the public, blocking gaps in windows or doors, or making changes to ventilation systems).

### Emergent theme: Learning and preparedness

Recipients reflected on how past forest fire experiences had been a source of learning, and how that learning could be applied to future forest fire seasons:

“*When [COMMUNITY A] evacuated to [COMMUNITY B]. That's how we found out that that happens. When they got to the, to the gyms, there was smoke coming in and that smoke, it kind of followed them from [COMMUNITY A], you know? And there was smoke coming in, so they had to block their ventilation system at those schools. And that's when we said, hey, this could happen to us too, back home like, so we prepared for that.*”

## Discussion

Report recipients expressed appreciation for “useful” and “critical” information specific to Eeyou Istchee delivered to them directly, and a desire for more visual elements and plain language. Future reporting seasons might therefore prioritize a shorter report with more visual elements (i.e., maps or infographics) or issuing a scaled-back report on days below thresholds of concern.

Smoke, air quality, and heat were identified as priority topics for informing public health response, while wind and visibility were useful in the context of impacts on existing fire(s) or smoke. When wind and visibility informed decision-making (i.e., evacuations, transportation convoys), these decisions required specificity beyond the scope of a daily Report at a regional level. To address this gap, one approach suggested by the evaluation team was to support recipients in seeking this information from trusted sources by continuing to include web links in the Report, holding training sessions, or developing guidance documents. Training sessions or tabletop exercises could also be used to address other emergent themes (i.e., the importance of jurisdictions and coordination in emergency response, applying learnings from past fires to future fires).

The Report was a source of reassurance among recipients with respect to their own and neighbouring communities, consistent with a lack of information being documented as a source of distress among First Nations people experiencing forest fires elsewhere in Canada (25). This expressed usefulness of the Report echoes the call for tailored communication and coordination strategies from trusted sources in the context of prolonged smoke and forest fire seasons (18,26,27).

This evaluation noted a number of challenges that are shared among jurisdictions in Canada, namely clarification of roles and responsibilities, and the need for specific public health guidance when smoke and heat occur simultaneously (28). In Canada, wildfire and heat health responses rely on centralized tools (e.g., AQHI and heat warnings) that emphasize standardization but may have limited local relevance and uptake (16,17). In contrast, this intervention (i.e. the Report) aligns with evidence from Indigenous and community-based emergency responses showing that culturally grounded, locally governed risk communication enhances trust, comprehension, and protective action during wildfire smoke, heat events, and other public health emergencies (18). Other challenges, such as summarizing and responding to conditions over a vast geographic area, are shared by Indigenous, rural, and remote jurisdictions (28–30).

While interviews focused on 2024 Report recipients, the distribution list expanded significantly in 2025, and additional recipients for 2026 and beyond (e.g., Elders’ homes) were identified during this article’s development. New recipients in 2025 were not included in key informant interviews, underscoring the importance of continuous evaluation and improvement.

### Limitations

This evaluation relied primarily on qualitative thematic analyses based on a small, purposive sample of key informants (n=6). Convenience sampling could introduce potential selection bias and incomplete capture of organizational perspectives, which constrains transferability. Analyses of public communications and temperature data were descriptive, temporally bounded, and selective in scope, which limits generalizability. Engagement metrics and forecast observation comparisons reflect proxy measures of reach and exposure and do not assess behavioural impact or other report components (e.g., air quality), representing additional sources of measurement bias.

## Conclusion

The *Eeyou Istchee Daily Smoke Monitoring Report* was considered useful and was a source of reassurance for recipients. Opportunities for improvement identified through this evaluation may also be relevant to other Indigenous organizations developing or enhancing their own environmental monitoring processes. These include the incorporation of additional visual elements, the use of plainer language, a streamlined report when conditions remain below thresholds of concern, and measures to help recipients access additional trusted information when needed for decision-making. Overall, the Report is a new health protection tool that complements the understanding Eeyouch/Eenouch have always had about living with fire as a natural force.

## Appendix

The Daily Smoke Monitoring Report included information on air quality, smoke forecasts, fire activity, health recommendations, temperature, wind and visibility. A detailed description of data elements, sources and rationale is available in the supplementary materials hosted on Open Science Framework (OSF). https://osf.io/nxewh/overview?view_only=cd44372ccee6452cb0d81d0938ed8b2a

Figure S1: Example of daily smoke monitoring report (2024)

Figure S2: Example of daily smoke monitoring report (2025)

Table S1: Data elements of the *Eeyou Istchee Daily Smoke Monitoring Report*

List S1: Conversation guide
